# Seasonal contributions of nutrients from small urban and agricultural watersheds in northern Poland

**DOI:** 10.7717/peerj.8381

**Published:** 2020-02-06

**Authors:** Karolina Matej-Lukowicz, Ewa Wojciechowska, Nicole Nawrot, Lidia Anita Dzierzbicka-Głowacka

**Affiliations:** 1 Faculty of Civil and Environmental Engineering, Gdansk University of Technology, Gdansk, Poland; 2 Marine Ecohydrodynamics Laboratory, Institute of Oceanology of Polish Academy of Sciences, Sopot, Poland

**Keywords:** Nutrients, Land use, Diffuse pollution, Urban catchment, Agricultural catchment, Baltic sea

## Abstract

Diffuse sources of pollution like agricultural or urban runoff are important factors in determining the quality of surface waters, although they are more difficult to monitor than point sources. The objective of our study was to verify assumptions that the inflow from agricultural nutrient sources is higher than from urbanized ones. It has been done by comparing the nutrients and organic matter concentrations and loads for three small streams in northern Poland (Pomerania Region). Two streams flowing through agricultural catchments and an urban stream flowing through the city of Gdansk were analysed. Concentrations of nutrients: N-NO${}_{3}^{-}$ N-NH${}_{4}^{+}$, P-PO${}_{4}^{3-}$, total phosphorus, total nitrogen and COD were measured 1–3 times per month in the period from July 2017 to December 2018 in agricultural watersheds and from October 2016 to March 2018 for an urban stream. Seasonal changes in concentrations were analysed with descriptive statistics tools. Principal Component Analysis (PCA) was used to point out the most significant factors determining variations in nutrients and organic matter concentrations with respect to different seasons. The factors included a number of characteristics regarding the catchment and streams: total catchment area, stream length, watershed form ratio, stream slope, flow rate and land use with respect to paved areas, agricultural areas and green areas (parks, forests, meadows and pastures). Although concentrations of nitrogen compounds were higher in streams flowing through agricultural areas, our study showed that total concentrations of phosphorus were higher in the urban stream, especially in summer. In agricultural areas the summer concentrations of nutrients were not high, which was probably due to dense vegetation. The correlation between P-PO${}_{4}^{3-}$ concentration and size of agricultural area in the catchment was observed in winter when no vegetation field cover exists. Our study shows an urgent need to monitor the nutrient loads carried with urban streams especially if discharged into receivers prone to eutrophication.

## Introduction

Surface waters undergo degradation as a result of anthropogenic activities in the watersheds, and this includes the consequences for agriculture, industry and urbanization. According to a UN report (United Nations, 2017) the intense population growth will continue beyond 2050 resulting in an increased demand for water and food production previously never experienced. The expanding cities will be home for more less 70% of the world’s population, resulting in increased impacts on surface and ground water resources through overcharging uptake and pollution, leading to water shortages, degradation of ecosystems, urban stream syndromes, reduction of biodiversity and contributing to climatic changes inside the cities (Zhao et al., 2013).

While addressing the pollution of surface waters, two major types of sources can be distinguished: point sources (e.g., municipal and industrial wastewater outflows, fish farming) and non-point or diffuse sources (runoff from agricultural or urban lands, atmospheric deposition) (Zieliński et al., 2016; Shoemaker, Ervin & DiOrio, 2017). Point sources are generally easy to monitor, while the diffuse pollution sources are usually more problematic to quantify and control. The types and loads of diffuse pollutants are strongly influenced by the land use. Runoffs from forests, meadows, agricultural lands and urbanized areas differ in their chemical composition (Pitt et al., 2004; Badruzzaman et al., 2012). River borne loads of nutrients discharged to marine waters contribute to (Iital et al., 2014; Øygarden et al., 2014; Nausch et al., 2017) the eutrophication of fresh waters as well as some susceptible marine waters like the inland, semi-closed Baltic Sea (Beutel et al., 2007; Fleming-Lehtinen et al., 2015; Padedda et al., 2017). Agriculture has been widely reported to be the major nitrogen supplier to river and marine waters (Iital et al., 2014; Øygarden et al., 2014; Nausch et al., 2017). Studies carried out in Finland and in the USA revealed that nitrogen input increased 8–10 times once a forested area had been transformed into agricultural land (Rekolainen et al., 1995; Hatfield & Follett, 2008). Kowalkowski et al. (2012) refers to the sum of tile drainage and groundwater as the “diffuse” load; according to these authors it represents approx. 31% of the P load discharged from the Polish territory to the Baltic Sea. According to HELCOM (2017), the riverine nutrient loads from Poland are characterised by comparatively large proportions from agriculture and from point-sources. For phosphorus, the point-sources share is even larger than the agricultural share (42%, and 34%, respectively). For nitrogen, the contrary occurs with 31% from point sources, and 45% from agricultural activities.

Both urbanization and intensive agriculture are major contributors of surface and ground water pollution, while the type and scale of water enrichment depends mainly on the land use structure, catchment characteristics and climate which influence the processes going on inside the catchment. Jekatierynczuk-Rudczyk et al. (Jekatierynczuk-Rudczyk, Zieliński & Górniak, 2006; Zieliński et al., 2016) analysed stream water quality in relation to land use in three types of watersheds, concluding that concentrations of dissolved organic carbon (DOC) in the agricultural watershed were two times higher than in the urban watershed and 6 times higher than in the forest catchment. These findings were explained by high ability of biomass production in the agricultural catchment. According to HELCOM the diffuse agricultural sources are the major contributor of nutrients delivered to the Baltic Sea, constituting approximately 40% of nitrogen and 30% of phosphorus (Sonesten et al., 2018). However, other types of pollutants are associated with urban catchments. For instance, heavy metals in agricultural catchments originate from pesticides, herbicides and mineral fertilizers (Yang & Toor, 2017; Xu et al., 2019), however urban and industrial sites contribute remarkably higher loads of this type of pollution (Szydłowski, Rawicki & Burczyk, 2017; Wojciechowska et al., 2019b). The increased concentrations of nutrients are also found in urban watersheds. Increase in impervious surfaces in urban watersheds results in elevated concentrations of N in storm water runoff (Wollheim et al., 2005; Kaushal et al., 2008) and a decline in biodiversity in streams (Paul & Meyer, 2001). Bao, Li & Cheng, (2018) reported increased deposition of phosphorus in the urban catchment, especially in retention tanks built on urban streams.

The average outflow of nutrients to the Baltic Sea from its watershed amounts to 17,698 m^3^/s, carrying 38,255 t of phosphorus and 976,941 t of nitrogen from agricultural and non-agricultural sources. Due to the specific geomorphological characteristics of the Sea, especially the limited water exchange through the Danish Straits, nutrient discharges at current levels are causing huge environmental problems and destabilizing the balance of the ecosystem, leading to harmful algal blooms, oxygen depletion and the formation of dead zones (Anderson et al., 2008; Heisler et al., 2008; Howarth, 2008; Smith & Schindler, 2009).

Among the Baltic states, Poland has the second largest area of agricultural lands while at the same time being the largest contributor of phosphorus load –8.6 kgP/ha of agricultural lands (Svendsen et al., 2015). Regarding nitrogen load, Germany holds the first place –107 kgN/ha, followed by Denmark 73 kgN/ha, Poland 70 kgN/ha and Finland 62 kgN/ha, while other countries discharge below 60 kgN/ha (Håkanson & Bryhn, 2008). Due to the Poland’s high position in this inglorious ranking, there is a need to focus close attention on nutrient inputs from Polish territory, not only in case of the large river watersheds that are usually carefully monitored, but also the smaller catchments too. Since Poland leads the list of phosphorus contributors in the region, the share of various sources in riverine catchments deserves a closer analysis.

The aim of the study was to compare the nutrient contribution with regard to land use (agricultural vs urban) as well as to distinguish the major factors determining nutrient concentrations in different seasons. The total annual loads of nutrients as well as aerial loads for each stream were calculated. Principal Component Analysis (PCA) was used to assess the factors influencing the nutrient concentrations in different seasons. The analysed factors included land use and a number of catchment and stream characteristics, including total catchment area, stream length, watershed form ratio, stream slope and flow rate.

## Materials and Methods

### Study area and sampling

Gdansk with almost half a million citizens, is the biggest city and capital of Pomeranian region and of northern Poland (Statistical atlas of Pomorskie Voivodship, 2018). The city area is expanding and urbanization, expressed as the proportion of paved and built-up areas, is rapidly increasing, putting more and more stress on surface waters due to increased volume of runoff transporting higher loads of pollutants washed out from the urban catchment.

In the current study, the contribution of nutrients from streams flowing to the Baltic Sea from the territory of northern Poland (Pomerania Province) was analysed. Three short, first-order streams with different land use were selected for analysis. The catchments of two streams (the Gizdepka (G) and Bladzikowski (BS)) are dominated by agricultural land use while the third (the Oliwski Stream (OS)) flows through forests in its upper part and through a densely urbanized area of Gdansk in its lower part.

In the current study three streams in the Pomerania region outflowing directly to the Baltic Sea (Gulf of Gdansk) were studied with respect to nutrient concentrations and loads carried. All streams are short (the length is below 10 km) which makes the self-cleaning processes less effective. The land use in the watersheds differs between the streams: two of them (the BS and the G) flow through typically agricultural areas with fields, meadows and pastures, while the third one—the OS—has its source in the forests surrounding Gdansk and then flows through some city districts, mostly in an open channel.

**Figure 1:**
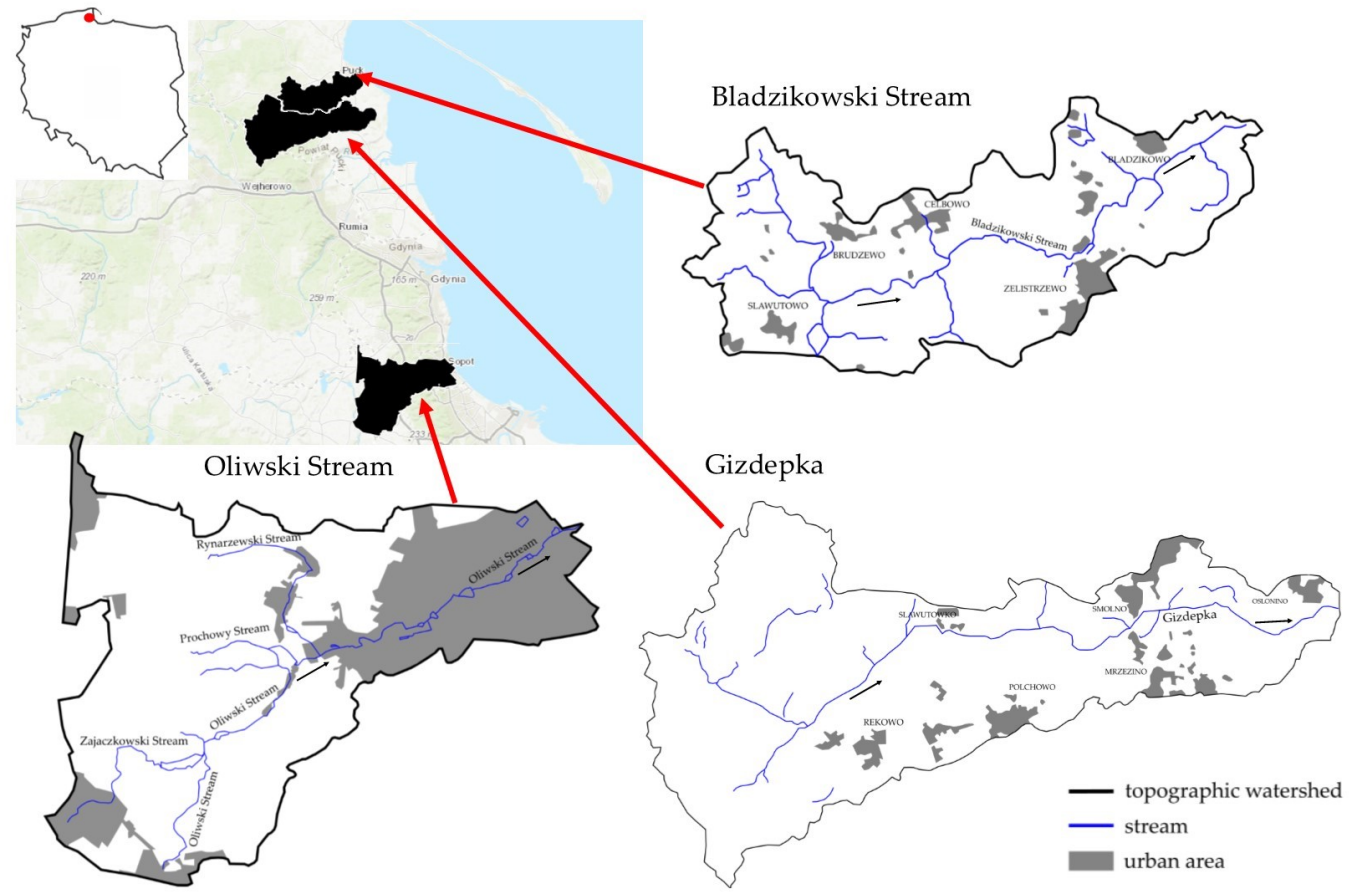
Location of streams and land use on the topographic map.

Two of the three analysed watersheds—the BS and the G—are located close to each other in the Puck Community (Pomerania Region). The watershed of the OS , flowing through the City of Gdansk, is located around 30 km south of the G watershed (Fig. 1). Table 1 summarizes the basic characteristics of two territory units: the City of Gdansk and Puck Community, where the analysed streams and their watershed are located.

**Table 1:** Basic data of municipalities where analysed watersheds are located.

**Parameter**	**Unit**	**City of Gdansk**	**Puck Community**
Population	[person]	464,254	26,018
Area	[km^2^]	262	237
Population density	[person/km^2^]	1,772	110
Share of the income from agriculture and hunting	[%]	0.6	2.7

Although the three analysed watersheds are located not far from each other, some small differences in climate do, in fact, exist including: average seasonal temperatures and rainfall, which are presented in Table 2. Higher temperatures (0.7 °C) are observed in the Puck Community in all seasons; the rainfall amounts are also higher there in each season apart from summer.

**Table 2:** Average temperatures and rainfall in different seasons in the City of Gdansk and Puck Community (The climate of Gdansk, 2018; The climate of Puck, 2018).

Average temperatures [°C]					
Gdansk	Spring	5.5	Puck	Spring	5.7
		Summer		15.4		Summer	16.2
		Autumn		7.7		Autumn	8.7
	Winter	−1.8		Winter	−0.9
The average amount of rainfall [mm]					
Gdansk	Spring	32	Puck	Spring	33
		Summer		66		Summer	65
		Autumn		49		Autumn	55
	Winter	33		Winter	39

The sampling points were located close to the discharge into the sea (Fig. 1). The sampling point on the BS was located 1.50 km from the mouth; the co-ordinates of the sampling point are 54.69582°N, 18.43652°E. The second sampling point on the G was approx. 0.60 km from the mouth (54.66441°N, 18.45897°E), while the sampling point on the OS was 0.1 km from the mouth (54.424691°N, 18.598976°E). The distance between first two points (BS and G) is 3.6 km in a straight line, while the last sampling point (OS) is located 32.0 km from BS and 28.2 km from G.

**Figure 2:**
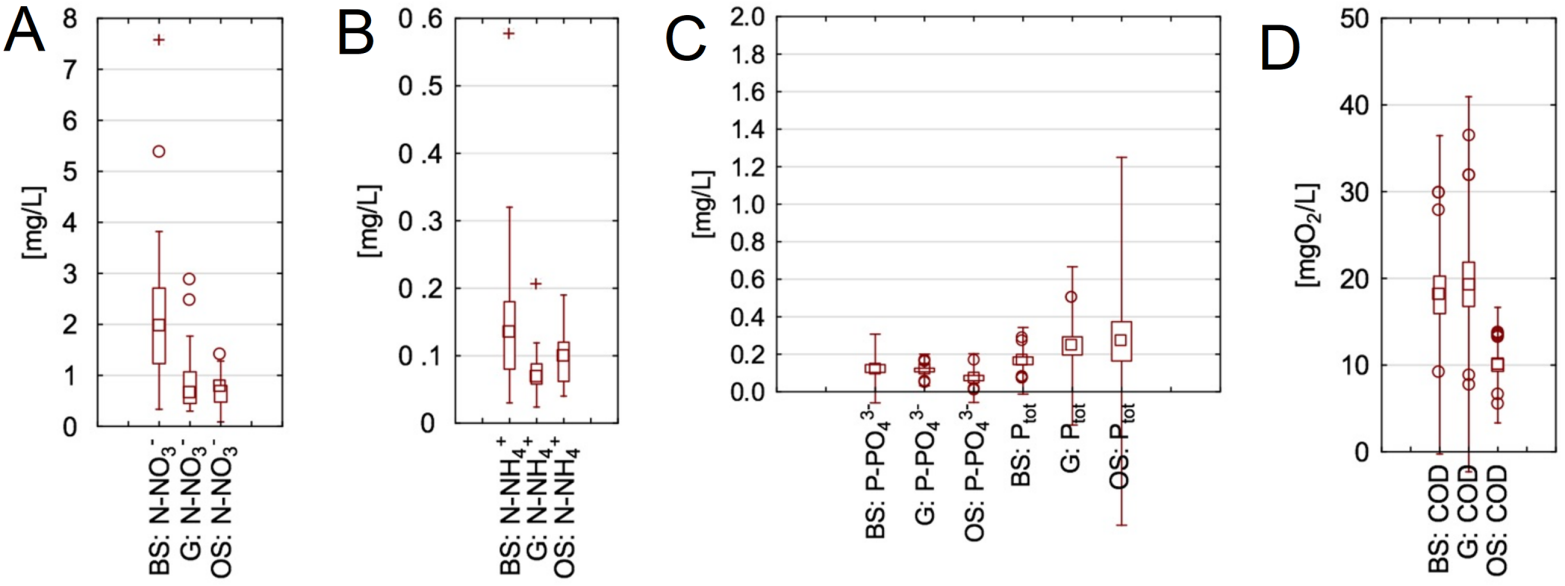
Concentrations of COD, nitrogen and phosphorus species in the analysed streams. (A) Concentration of NO_3_^−^, (B) concentration of NH_4_^+^, (C) concentration of P-PO_4_^3−^ and Ptot, (D) concentration of COD. BS, the Bladzikowski Stream; G, the Gizdepka; OS, the Oliwski Stream.

**Figure 3:**
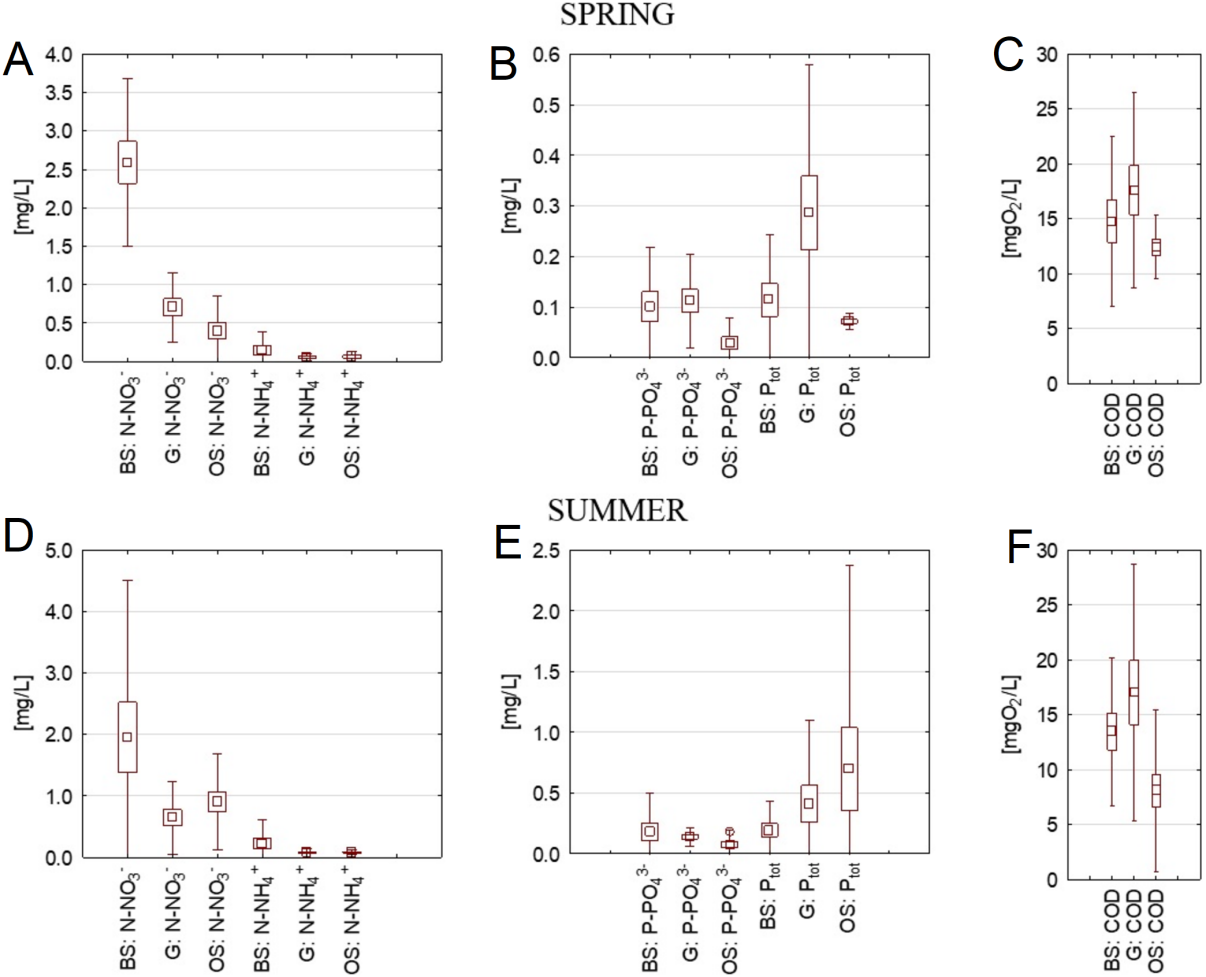
Concentrations of nutrients and COD in cross-sections of three streams in spring and summer. (A) Concentration of NO_3_^−^, and NH_4_^+^ in spring, (B) concentration of P–PO_4_^3−^ and Ptot in spring, (C) concentration of COD in spring, (D) concentration of NO, and NH in summer, (E) concentration of P–PO_4_^3−^ and Ptot in summer, (F) concentration of COD in summer. BS, the Bladzikowski Stream; G, the Gizdepka; OS, the Oliwski Stream.

The Bladzikowski Stream drains the eastern part of Kepa Swarzewska (Swarzewo Elevation) while the Gizdepka drains the southern part of Kepa Pucka (Puck Elevation). The watersheds have a dense network of channels and drainage ditches regulating the water level to obtain optimal conditions for agriculture. In the analysed area two aquifers are present: the Tertiary at a depth of 5–10 m below ground and the Quaternary at a depth of 20–50 m below ground. Potrykus et al. (2018) and Szymkiewicz et al. (2018) calculated the infiltration time of groundwater (for the Tertiary aquifer) in the area, the results varied between 257 and 15,261 days. The land use of both watersheds based on GIS maps (Figs. 2 and 3) and the questionnaire responses of selected representative farms (Pietrzak, 2018; Dzierzbicka-Glowacka et al., 2019; Wojciechowska et al., 2019c) is presented in Table 3.

**Table 3:** Parameters of streams and catchments (Jereczek-Korzeniewska, 2010; Zima, 2019).

Characteristics	Symbol	Description	Unit	Oliwski Stream	Gizdepka	Bladzikowski Stream
Area	A	Surface area of the watershed [km^2^]	[km^2^]	28.92	37.20	23.00
Length	L	Length from source to the discharge (mouth) [km]	[km]	9.6	13.6	11.0
Watershed form ratio	F	Area to length ratio [km^2^/km]	[km^2^/km]	3.0	2.7	2.1
Mean slope of the bed	d_p_	The ratio between height difference between the stream source and stream discharge (mouth) and stream length [‰]	[‰]	14.6	7.8	4.0
Paved area	Ap	Surface area of paved lands [km^2^]	[km^2^]	12.34	2.15	1.54
Agricultural area	Aa	Surface area covered with fields [km^2^]	[km^2^]	0	12.00	16.68
Area covered with meadows, pastures and parks	Ag	Surface area covered by meadows, pastures, parks [km^2^]	[km^2^]	16.58	23.05	4.78
Flow rate	Q	The mean flow rate in the stream [m^3^/s]	[m^3^/s]	0.21	0.17	0.04

The OS flows through northern part of Gdansk. In the Pomerania region 64.2% of the population lives in a city. The OS has its source in the Tricity Landscape Park and the upper part of the catchment is covered by forests. In the middle and lower run, the Stream flows through residential areas with single-family and multi-family houses, receiving the runoff from parking lots, roads, roofs and park areas. The lower part of the catchment is being intensely developed and every year new housing estates and paved areas spring up there. The discharge of the OS to the Gulf of Gdansk is located at a small distance to the popular beach and bathing place. The OS mostly flows in an open channel; there are only a few intersections with roads where it is covered.

The analysed catchments were characterized by the 9 parameters listed in Table 3. In Table 3 the values of the parameters for each of the streams and watersheds are presented. The data presented in Table 3 were determined based on the analysis of maps provided by the Puck Commune and the values given by Jereczek-Korzeniewska (2010) and Zima (2019).

Among the analysed streams, the G has the largest catchment area and length. The OS has the highest watershed form ratio (area per unit length). The OS also has the highest bottom slope, resulting in a faster concentration of the runoff during rainfall events. The flow rate of the BS is only 0.04 m^3^/s; for the G it is four times higher while for the OS it is almost six times higher than in the BS.

### Methodology

Samples were collected with a frequency of 1–3 samples per month. The sampling period lasted from July 2017 to December 2018 for the BS and the G and from October 2016 to March 2018 for the OS. In total, 50 samples were taken from each stream. The analysis was carried out in a laboratory at Gdansk University of Technology. The measurements of the concentrations of nitrates (V) (N-NO_3_), ammonium nitrogen (N-NH_4_), orthophosphates (P-PO_4_), total phosphorus (Ptot), total nitrogen (Ntot) and COD were carried out using spectrophotometric methods based on the Hach cuvette tests. The nitrate concentrations (V) were measured according to EN 38405 D-2, ammonia nitrogen – ISO 7150-1, DIN 38406 E5-1, UNI 11669: 2017, phosphate phosphorus and total phosphorus – EN ISO 6878, total nitrogen - EN ISO 11905-1 and COD according to PN-74/C-04578/03. The measurements were performed with a VIS DR3900 spectrophotometer Hach, and the HT200S Hach thermostat was used for mineralization. The tests were carried out in three replicates for each sample and the final result is an arithmetic mean. Total nitrogen was measured in BS and G, but it was not measured in PO.

The load of nutrients and organic matter discharged by the three analysed streams was calculated based on concentration measurements (2–3 measurements per month) and the flow rate measured while sampling. In each point the geodetic measurement of the stream cross-section was made, which allowed for determination of the cross section area after each change of the water level. Additionally, the flow velocity was measured during each sampling. Those two parameters allow for determination of the flow rate. The flow rate was determined by using hydrometric plumbs (Flo-Mate 2000 produced by Marsh McBirney). The monthly loads were described and the annual loads (*L*_*a*_) were calculated using the monthly loads (*L*_*m*_*i*__).

The average daily load (*L*_*d*_*i*__) in each month was calculated according formula (1) and the monthly load (*L*_*m*_*i*__) according to formula (2). The average annual load (*L*_*a*_) was calculated as a sum of monthly loads. Apart from flow measurements, geodetic measurements were also performed at each cross-section.

(1) \begin{eqnarray*}{L}_{{d}_{i}}& = \frac{\sum _{1}^{n}{Q}_{i}\cdot {c}_{i}}{n} \left[ \frac{mg}{day} \right] \end{eqnarray*}

(2) \begin{eqnarray*}{L}_{{m}_{i}}& ={L}_{{d}_{i}}\cdot m \left[ \frac{mg}{month} \right] \end{eqnarray*}

(3) \begin{eqnarray*}{L}_{a}& =\sum _{1}^{12}{L}_{{m}_{i}} \left[ \frac{mg}{a} \right] \end{eqnarray*}

where:

n, number of measurements per month,

Q_i_, instantaneous flow rate [L/day],

c_i_, instantaneous concentration [mg/L],

m, number of days in a month.

Statistical analysis of the results was carried out using the Statistica® 13.1. Descriptive statistics and analysis of the main components were used. Principal Component Analysis was used to determine the relationship between the variables.

## Results

### Mean concentrations

In Fig. 2 the ranges of concentrations of nutrients and COD during the whole period of analyses are presented. The highest concentrations of nitrogen compounds (N-NO${}_{3}^{-}$ and N-NH${}_{4}^{+}$) were measured in the BS. The COD concentrations were highest in G, although the concentrations in the BS were only slightly lower. In turn, the highest total phosphorus concentrations were present in the OS. The ranges of all measured compounds were high, which is shown on the box plots.

### Annual loads and land Use

The annual loads of nutrients discharged by the analysed streams are presented in Table 4. Additionally, the aerial loads (the ratio of pollutant loads to the watershed area) were calculated for the watersheds of all streams and the results are presented in Table 5.

**Table 4:** Annual loads discharged to the Baltic Sea by the analysed streams [t/year].

Stream	N-NO${}_{3}^{-}$	N-NH${}_{4}^{+}$	Ntot	P-PO${}_{4}^{3-}$	Ptot	COD
Bladzikowski Stream	2.57	0.17	4.04	0.14	0.18	20.0
Gizdepka	5.06	0.42	6.06	0.63	1.34	105.4
Oliwski Stream	4.54	0.68	–	0.48	1.75	65.0

**Table 5:** Annual loads in relation to the catchment in cross-sections at the estuary of the Baltic Sea [kg/ year km^2^].

Stream	N-NO${}_{3}^{-}$	N-NH${}_{4}^{+}$	Ntot	P-PO${}_{4}^{3-}$	Ptot	COD
Bladzikowski Stream	0.11	0.007	0.18	0.006	0.01	0.9
Gizdepka	0.14	0.011	0.16	0.015	0.04	2.8
Oliwski Stream	0.16	0.023	–	0.016	0.06	2.2

The highest load of nitrates (V) was discharged by the G (the stream flowing through agricultural and forested areas). However, in terms of loads per catchment area, the highest load was discharged by the OS flowing through an urban catchment (>42% of the catchment is urbanized). Also the ammonia nitrogen loads (total and aerial) were highest for an urban stream. The highest load of phosphates was discharged by the G, while the lowest was by the BS (with agricultural fields, meadows and pastures occupying almost 90% of the catchment area).

Comparing the aerial loads of P-PO${}_{4}^{3-}$, the values for the G and the OS were quite similar. The total phosphorus loads per year were again highest in the case of the OS, and lowest for the BS. The highest annual loads of organic matter (COD) were discharged by the G, which had a high share of forests in its catchment (<40%).

The concentrations of total nitrogen in Błądzikowski Stream were approximately 3 times higher than in Gizdepka (the median in BS was equal to 3.20 mg N/L while in G it was 1.13 mg N/L). Nitrates had the largest share in the load of total nitrogen – 64% for Błądzikowski Stream and 83% for Gizdepka.

Surprisingly, the watershed of the OS (mostly urban) turned out to have the highest aerial loads of four out of five examined pollutants: N-NO${}_{3}^{-}$, N-NH${}_{4}^{+}$, P-PO${}_{4}^{3-}$ and Ptot. This finding was different from previous observations, where mainly the increase in nutrient concentration in the Baltic countries was analysed, inter alia due to the extended vegetation period caused by global warming (Iital et al., 2014; Øygarden et al., 2014; Nausch et al., 2017). Comparison of the loads of nutrients and COD discharged by analysed streams to the Baltic Sea showed that despite the high concentrations, the loads carried by the BS were not so high as other streams, due to its low flow rate.

### Seasonal changes

The average yearly concentrations and loads of nutrients and organic matter give some general idea of the scale of pollution discharged into the Baltic Sea, showing differences between the streams of differently used watersheds. We also analysed the seasonal patterns and differences between agricultural and urban watersheds.

In spring, the highest concentrations of nitrogen species were present in the BS with mean concentrations of N-NO${}_{3}^{-}$ and N-NH${}_{4}^{+}$ even 7 times higher than in the OS. The highest concentrations of phosphorus compounds occurred in the G, over 3 times higher than in the OS. Concentrations of organic matter (COD) in the G and the BS were at a similar level, higher than in the OS.

In summer, concentrations of P-PO4^3−^, N-NO${}_{3}^{-}$ and N-NH${}_{4}^{+}$ were again highest in the BS. Total phosphorus concentrations were highest in the OS, almost 2 times greater than in the G and 4 times higher than in the BS. COD concentrations were higher in the G and the BS than in the OS, similarly as in spring.

In autumn, the valuesregarding nitrogen compound concentrations were similar to spring and summer. Small discrepancies between phosphorus compound concentrations were observed, with average concentrations slightly higher in BS and G than in OS. COD concentrations were again higher in BS and G.

In winter, concentrations of N-NO${}_{3}^{-}$ were over 3 times higher in the BS than in the OS and N-NH${}_{4}^{+}$ concentrations were 2 times higher in BS than OS. Average concentrations of P-PO${}_{4}^{3-}$in all streams were at a similar level, although the highest variation range occurred in the OS. Concentrations of Ptot decreased in the following order: BS, G, OS. COD concentrations remained higher in the streams flowing through agricultural watersheds than in OS.

To summarize, spring concentrations of all analysed compounds were higher in streams with agricultural watersheds. Concentrations of nitrogen compounds and organic matter were higher in streams with an agricultural catchment than in the OS in all seasons. Total phosphorus concentrations in summer were highest in the OS. The highest yearly Ptot concentrations calculated for the OS resulted from high summer concentrations.

**Figure 4:**
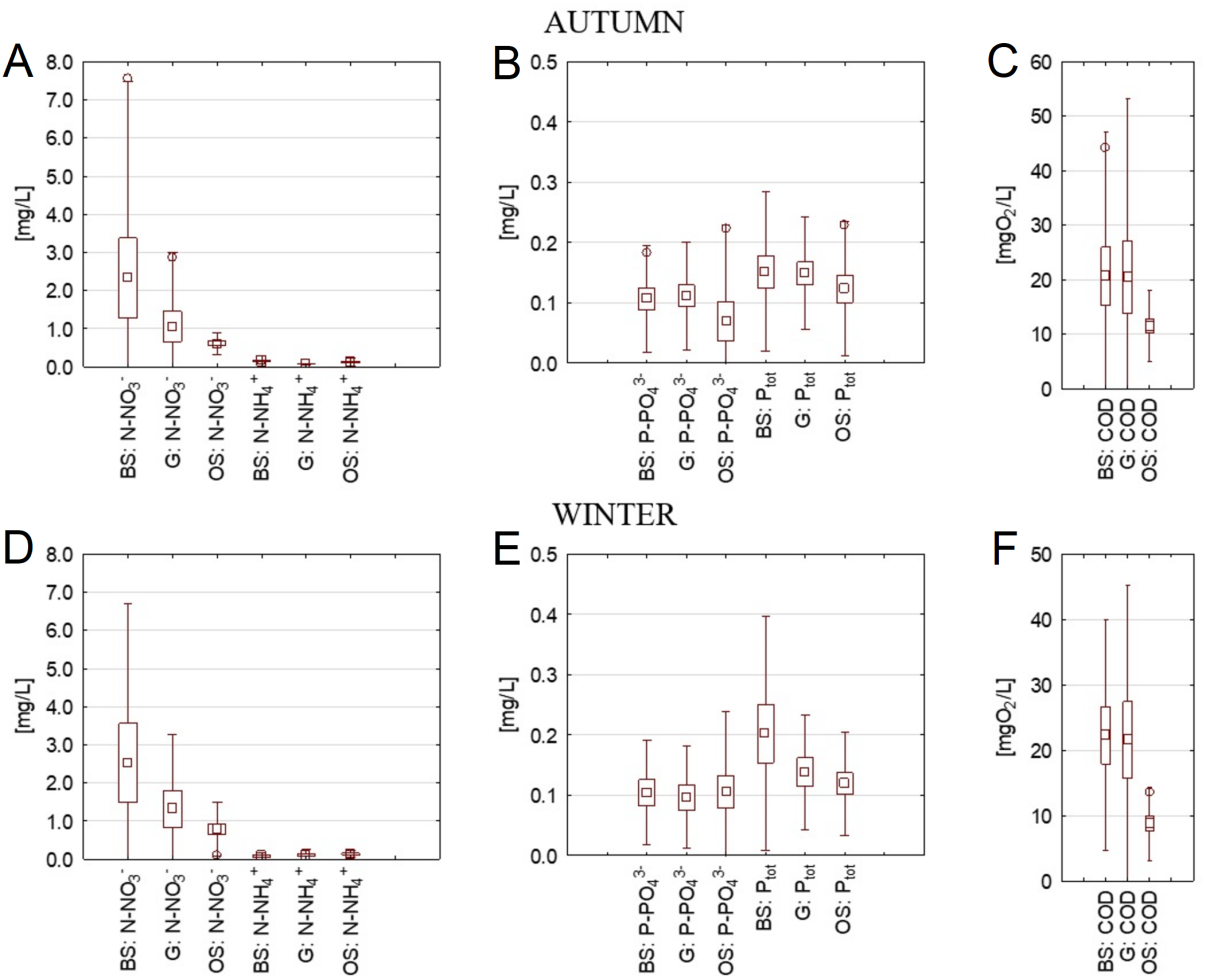
Concentrations of nutrients and COD in cross-sections of three streams in autumn and winter. A) Concentration of NO_3_^−^, and NH_4_^+^ in autumn, (B) concentration of P-PO_4_^3−^ and Ptot in autumn, (C) concentration of COD in autumn, (D) concentration of NO_3_^−^, and NH_4_^+^ in winter, (E) concentration of P-PO_4_^3−^ and Ptot in winter, (F) concentration of COD in winter. BS, the Bladzikowski Stream; G, the Gizdepka; OS, the Oliwski Stream.

Analysis of maximal concentrations showed that all maximum concentrations occurred in the OS (in summer: N-NH${}_{4}^{+}=0.36$ mg/L, P-PO${}_{4}^{3-}=0.22$ mg/L, Ptot = 0.42 mg/L, in autumn: N-NO${}_{3}^{-}=4.42$ mg/L and COD = 36.0 mgO_2_/L). The out-of-scale values were not shown on Figs. 3 and 4.

The results presented in Table 6 were used to evaluate the trends of seasonal changes in different compound concentrations in the streams analysed. In the OS, a summer increase in all nutrient concentrations was observed; in case of N-NH${}_{4}^{+}$ this elevation lasted until winter. The organic matter concentrations varied they increased in spring and autumn but decreased in summer and winter. In the BS, a summer elevation of concentrations of phosphorus compounds and N-NH${}_{4}^{+}$ was noted, while the concentration of N-NO${}_{3}^{-}$ increased between spring and autumn; COD concentrations were higher in autumn and winter than in summer. Concentrations of phosphorus compounds increased in the G in summer, yet then decreased in autumn and winter. Concentrations of N-NH${}_{4}^{+}$ increased subsequently in spring, summer and autumn, while COD increased in autumn and winter.

**Table 6:** Trends of seasonal changes of COD, nitrogen and phosphorus species concentrations.

Parameter	N-NO${}_{3}^{-}$	N-NH${}_{4}^{+}$	P-PO${}_{4}^{3-}$	P_tot_	COD
Unit	[mg/L]				[mgO_2_/L]
Oliwski Stream					
Su/Sp	↑	↑	↑	↑	↓
A/Su	↓	↑	↓	↓	↑
W/A	↑	↑	↑	↓	↓
Sp/W	↓	↓	↓	↓	↑
Błądzikowski Stream					
Su/Sp	↓	↑	↑	↑	↓
A/Su	↑	↓	↓	↓	↑
W/A	↑	↓	↓	↑	↑
Sp/W	↑	↑	↓	↓	↓
Gizdepka					
Su/Sp	↓	↑	↑	↑	↓
A/Su	↑	↑	↓	↓	↑
W/A	↑	↑	↓	↓	↑
Sp/W	↓	↓	↑	↑	↓

**Notes.**

Su/Sp change in summer-spring period A/SU autumn/summer W/A winter/autumn Sp/W spring/winter ↑ increased concentration ↓ decreased concentration

A generally observed trend was an increase in N-NH${}_{4}^{+}$ and phosphorus compound concentrations in summer, regardless of the type of watershed (agricultural vs urban). In streams flowing through agricultural watersheds, concentrations of N-NO${}_{3}^{-}$ and COD increased in autumn and winter. In BS, with agricultural land use of the watershed dominating, the increase in Ptot concentration occurred in winter and N-NO${}_{3}^{-}$ concentrations elevation was observed in spring. In the G, where the watershed is partly covered by forests, meadows and pastures, concentrations of all compounds (apart from organic matter) decreased only in winter.

### Principal component analysis

**Figure 5:**
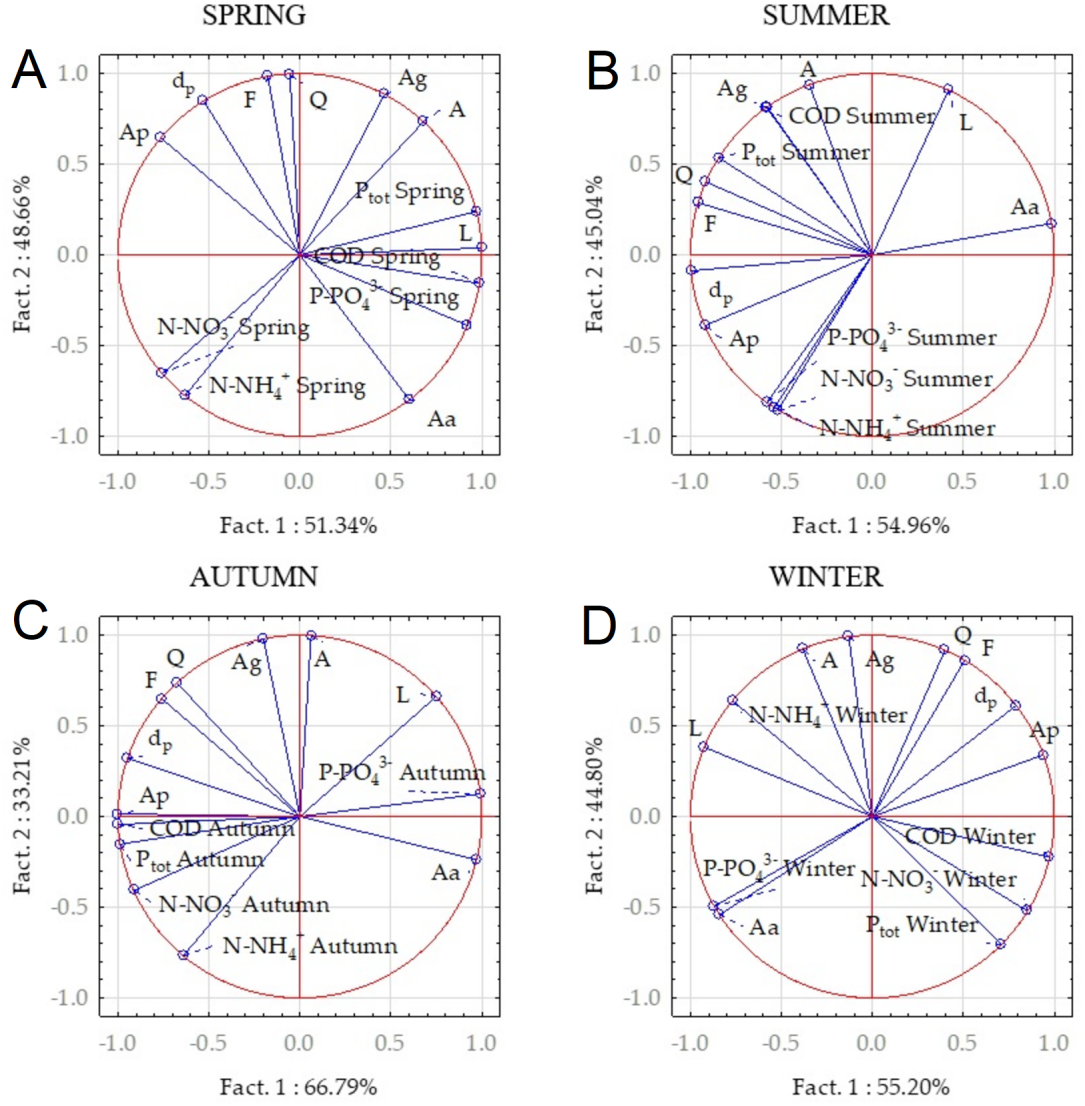
Relationships between concentrations of analysed compounds and catchment characteristics in different seasons derived from PCA. (A) Spring, (B) summer, (C) autumn, (D) winter.

Prinicpal Component Analysis was used to carry out a quantitative assessment of how a number of catchment area characteristics influence the quality of streams. The results of the analysis in relation to seasons are presented in Fig. 5.

In each season the new variables were determined that replaced the 13 initial (primary) variables. The initial variables were very well represented by the new ones, which is confirmed by the length of the vectors showing the primary variables were equal to unity. The new variables for each season represent 100% of the initial variables.

In spring the concentrations of COD, P-PO${}_{4}^{3-}$ and Ptot were related to the stream length (L) and reversely co-related to the urbanized area (Ap) and the stream slope (dp). Additionally, P-PO${}_{4}^{3-}$ concentration is positively co-related to agricultural area (Aa). The changes in N-NO${}_{3}^{-}$ and N-NH${}_{4}^{+}$ concentrations were negatively related to the total area of the catchment (A) and area covered by parks, forests and pastures (Ag).

In summer, concentrations of Ptot and COD depended on the amount of green area (Ag) and total catchment area (A). This can be explained by runoff pollution with animal excrement (Pitt et al., 2004). Ptot concentration was also related to the flow rate (Q) and form ratio (F). P-PO${}_{4}^{3-}$, N-NO${}_{3}^{-}$ i N-NH${}_{4}^{+}$ have a common origin co-related to the size of urbanized area (Ap); all of these factors were negatively related to stream length (L).

High summer concentrations of all analysed compounds observed in an urban stream result from the land use of the catchments. In agricultural watersheds, an increase in nutrient concentrations was not observed probably due to vegetation cover on the fields and meadows, followed by nutrient uptake by the intense growth of plant biomass. Water uptake by plants and infiltration through permeable soil (Yang et al., 2016) resulted in smaller runoff volume and thus a lower amount of nutrients in the streams.

In autumn, concentrations of Ptot and N-NO${}_{3}^{-}$ were related to the paved area (Ap) and catchment slope (dp). Concentrations of phosphate phosphorus were related to agricultural area (Aa). The stream characteristics length, flow rate, catchment form ratio were less significant than in other seasons.

In autumn and winter, a decrease in phosphorus concentrations was expected, and in fact did occur throughout the majority of these seasons. Some peaks in concentrations can be associated to the release of phosphorus compounds from the sediments found in retention tanks, as was reported by Bitschofsky & Nausch (2019) in the Warnow catchment in Germany. The autumn increase in N-NO${}_{3}^{-}$ concentrations in comparison to the spring period, observed only in agricultural watersheds, was probably caused by washing out the fertilizing compounds applied to the fields earlier in autumn.

In winter, concentrations of organic matter (COD) were positively co-related to urbanized area (Ap) and also to N-NO${}_{3}^{-}$ and Ptot concentrations. Concentrations of P-PO${}_{4}^{-}$ in this season were related to agricultural area (Aa). Probably the phosphates added to the soil with autumn fertilization are washed out in the winter period as the soil has no vegetation cover (Wojciechowska et al., 2019a); also some additional winter supply of fertilizers could be the reason. According to the questionnaire conducted in the agricultural catchments, some farmers indicated that they also use fertilizers in November, December and January (Pietrzak, 2018). Concentrations of N-NH${}_{4}^{+}$ in winter were dependent on the total catchment area (A) and stream length (L).

Separate observations were carried out in each of the seasons, which, however, made it possible to systematize the main parameters affecting the concentrations of the analyzed compounds. Total phosphorus concentrations depended on catchment parameters from spring to autumn. In spring and summer these were the length, flow rate, catchment shape factor or its slope, while in autumn paved area was more important. Nitrogen (N-NO${}_{3}^{-}$ and N-NH${}_{4}^{+}$) concentrations in summer and autumn depended mainly on the area of urbanized land, only in winter the entire catchment area and the length of the watercourse were important. However, the COD concentration was associated with the method of covering the catchment area, with the most important being the area covered with meadows, pastures and parks in summer and the paved area in autumn.

Principal component analysis indicated the origin of phosphorus compounds (P-PO${}_{4}^{-}$ and Ptot) in every season of the year. Spring was the only season when both forms of phosphorus were associated with a common source: the urbanized area and stream slope. Concentration of P-PO${}_{4}^{-}$ was also linked to the agricultural area; this correlation was strongest in winter, but also relevant in spring and autumn. PCA was also helpful to some extent in explanation of nitrogen species origin (N-NO${}_{3}^{-}$ and N-NH${}_{4}^{+}$). In spring and summer the reverse relationships were observed between nitrogen forms concentrations and total area as well as area covered by meadows, pastures and parks. In autumn the N-NO${}_{3}^{-}$ concentration was dependent on Ap and dp, while in winter the N-NH${}_{4}^{+}$ concentration was related to A and L. The lowest significance of 9 analysed stream and watershed parameters had form ratio, flow rate and length.

## Discussion

According to Valle et al. (2014) and Pacheco & Fernandes (2016) the main reason for the deterioration of surface water quality are changes and environmental land use conflicts. Shi et al. (2017) underlined that both intensive agricultural use and rapid urbanization have a destructive effect on water quality.

In our study, the expected higher concentrations of analysed compounds in streams in agricultural catchments (BS, G) were confirmed in the case of nitrogen compounds and organic matter. However, total phosphorus concentrations were higher in the OS with an urbanized watershed. Tufford et al. (2003) who investigated streams in coastal region (USA), also reported higher concentrations of N-NH${}_{4}^{+}$ in streams with forested watersheds, while concentrations of N-NO${}_{3}^{-}$ and Ptot were higher in streams with urbanized watersheds.

In our study, the loads of pollutants were higher for the urban stream, mostly due to a higher flow rate. On the other hand, it was proved that the flow rate increases with the progress of urbanization (Oudin et al., 2018); at the same time the retention capacity of the catchment decreases (Kanclerz et al., 2016). The comparative analysis of concentrations and loads of pollutants with regard to land use of the watershed was performed in similar way as in the study by O’Driscoll et al. (2010).

Pratt & Chang (2012) as well as Chen et al. (2018) analysed seasonal changes of nutrients in surface waters, reporting TSS and Ptot concentrations increase in dry season (June-October). Similarly, in the catchments analysed in this study, an increase in the total phosphorus concentration was observed in the spring-summer period. The previously mentioned authors also noticed an increase in N-NH${}_{4}^{+}$ and N-NO${}_{3}^{-}$ concentrations from September to March in China. This was not confirmed entirely in the analysed catchments in Pomerania, however, an increase in N-NH${}_{4}^{+}$ concentration was observed from summer to winter in OS and G and also in all streams concentration of N-NO${}_{3}^{-}$ increased from autumn to winter. Investigations carried out on the river Sava flowing through Slovenia, Bosnia and Hercegovina and Serbia indicated that N-NO${}_{3}^{-}$ concentrations were highest in winter and lowest in summer due to intense uptake by phytoplankton (Vrzel et al., 2016). A similar tendency occurred in the Gulf of Gdansk (SATBALTYK, 2018). Tufford et al. (2003) reported that concentrations of N-NH${}_{4}^{+}$, Ntot and Ptot were higher in summer, while N-NO${}_{3}^{-}$ concentrations peaked in winter.

Lin et al. (2019) noted that in spring N-NO${}_{3}^{-}$ was released from agricultural sources while in summer and autumn this inflow decreased. This is evidently related to agricultural practices that are determined by climate and the type of plants cultivated, and so vary between different parts of the world. In the current study the inflow of N-NO${}_{3}^{-}$ in spring occurred in the case of the BS, which was clearly related to the highest share of agricultural fields in its watershed, and the use of mineral fertilizers that were washed away from the fields to the drainage ditches and canals contributing to the stream draining the whole area (Wojciechowska et al., 2019a). The autumn and winter increase in N-NO${}_{3}^{-}$ concentrations observed in the BS and in the G could be a result of negligible consumption by plants and washing out of minerals after autumn fertilization (Lin et al., 2019).

O’Driscoll et al. (2010) proved that the urbanization of agricultural and forested areas results in increased oxygen demand, conductivity, concentrations of TSS, nutrients and heavy metals. According to Schuler et al. (Schueler, Fraley-McNeal & Cappiella, 2009) and Moring (2009) the key factor causing the deterioration of water quality in urban streams is runoff from urbanized areas.

The elevated summer concentration of total phosphorus in the OS, similar to the result gained during the investigations of Tufford et al. (2003), may origin from 13 retention tanks located on the stream. According to Bao, Li & Cheng (2018) phosphorus captured in sediments at the bottom of tanks can be released back to the water column—the phosphorus cycle depends, among other, on redox potential, temperature, pH, Fe and Mn concentrations. Another explanation of the summer peak phosphorus concentration in an urban stream could be the increased runoff caused by high summer rainfall, as the summer rainfalls constitute the highest sum in the total yearly rainfall amount in Poland (Table 2) (Lin et al., 2019).

Analysis of pollutant origins confirmed that season, type of stream as well as catchment characteristics are important controlling factors (Tufford et al., 2003; Moring, 2009; O’Driscoll et al., 2010; Kanclerz et al., 2016; Chen et al., 2018). Previous studies have used the analysis of Pearson’s correlation (Yu et al., 2016) or Spearmana’s correlation (Tu, 2011), in this study a method was used to examine the correlation between a larger number of variables–Principal Component Analysis. PCA was used in our study to distinguish the factors responsible for water quality changes. The results for different seasons are presented in Fig. 5. Similar analyses were previously performed by Xu et al. (2019), while Yang & Toor (2017) used PCA to evaluate the major factors that determine water quality changes without seasonal effects.

Areas covered with meadows, pastures and parks (Ag) are considered to be the main factor limiting contamination with nutrients, which is the result of the capability of plants for nutrient absorption and assimilation and also to decrease soil erosion (Li et al., 2018). It has been observed that spring concentrations of N-NO${}_{3}^{-}$ andN-NH${}_{4}^{+}$ decreased as the area of Ag increased. In summer, in addition to nitrogen compounds, concentrations of P-PO${}_{4}^{3-}$ decreased as the area of Ag increased. In autumn, organic material (COD) was also included in this group. In winter, the ability of nutrient absorption by plants decreases, therefore, the above relationships were not observed.

Self-purification is a complex chemical, biological and physical process (Mbuligwe & Kaseva, 2005) that is influenced by the parameters of the watercourse, such as length, bottom drop or type of channel (artificial, natural). The three analysed streams are relatively short, but they have natural channels, so the process of how the adsorption of contaminants on the surface of the bottom and edges was more effective.

## Conclusions

Comparison of the loads of nutrients discharged from the catchment with different land use showed that higher nitrogen loads were released from agricultural catchments while the urban catchment discharge higher load of phosphorus. The differences were higher in summer than in other season. This can be explained by the fact that dense summer vegetation in agricultural cover prevents phosphorus loss through erosion. In terms of loads per catchment area, the highest loads of nutrients (N-NO${}_{3}^{-}$, N-NH${}_{4}^{+}$, P-PO${}_{4}^{3-}$ and Ptot) were discharged by the urban stream. Only organic matter concentration expressed in COD were higher in stream with agricultural catchments than in the urban stream.

With regard to seasonal effects, our study confirmed the relationship between urbanized areas in the catchment and concentrations of nutrient compounds and organic matter in summer and autumn. Surprisingly, in agricultural areas, the runoff from fields in summer did not cause deterioration of stream water quality, which was most likely due to dense vegetation.

PCA including nutrient concentrations and watershed and stream characteristics was more helpful in explanation of phosphorus compounds than nitrogen compounds and organic matter origin. The analysis of factors that influence nutrient concentrations in the analysed watercourses showed that the green area size in the catchments played the most relevant role, while stream length, flow rate and watershed form ratio were of the lowest significance Both nitrogen and phosphorus concentrations were low in the catchments with larger share of green areas.

The expansion of urban areas is likely to cause a continuous increase in flow rates in urban streams and, as indicated in this study, higher aerial loads of nitrogen and phosphorus compounds. This indicates an urgent need to monitor the nutrient loads carried by urban streams, especially if discharged into receivers prone to eutrophication, such as the Baltic Sea and to develop mitigation strategies particularly fitting in the urban landscape The higher proportion of green areas acting like buffer zones to mitigate diffuse pollution is supported by the results of our study.

##  Supplemental Information

10.7717/peerj.8381/supp-1 Supplemental Information 1 Raw data

## Additional Information and Declarations

### Data Availability

The following information was supplied regarding data availability: The raw measurements are available as a Supplemental File.
